# Orbital intramuscular hydatid cyst causing compressive optic neuropathy: a case report and literature review

**DOI:** 10.1186/s12886-024-03502-w

**Published:** 2024-06-14

**Authors:** Ali A. Haydar, Seyed Mohsen Rafizadeh, Elham Rahmanikhah, Zohreh Nozarian, Amirhossein Aghajani, Mohammad Taher Rajabi

**Affiliations:** 1 Department of Orbital and Oculoplastic Surgery, Farabi Eye Hospital, Tehran University of Medical Sciences, South Kargar Street, Qazvin Square, Tehran, 1336616351 Iran; 2 Department of Ophthalmic Pathology, Farabi Eye Hospital, Tehran University of Medical Sciences, Tehran, Iran

**Keywords:** Echinococcus, Endemic, Hydatid cyst, Orbit, Optic neuropathy, Zoonotic

## Abstract

**Background:**

Echinococcosis, commonly known as hydatid disease, is a zoonotic infection resulting from the tapeworm *Echinococcus granulosus*. The occurrence of hydatid cysts in the orbital region is uncommon, representing less than 1% of all reported hydatid cases. This report details a unique case of an intramuscular hydatid cyst in the orbital region that led to compressive optic neuropathy.

**Case Presentation:**

A 22-year-old male from Kabul, Afghanistan presented with a five-month history of progressive proptosis in his left eye, associated with a gradual decrease in vision over the past three weeks. The left eye exhibited upward globe dystopia, ocular motility limitation, mild conjunctival injection, and chemosis. Diagnosis was achieved through imaging and histopathological examination. Treatment involves surgical removal of the cyst and prolonged albendazole therapy. The postoperative course showed significant improvement in the patient’s condition and restoration of his vision.

**Conclusions:**

Despite its rarity, this case underscores the importance of awareness and knowledge of hydatid disease among physicians, especially those working in endemic areas. It emphasizes the importance of including hydatid disease in the differential diagnosis of orbital masses, particularly in endemic regions.

## Background

Hydatid disease, also known as echinococcosis, is a zoonotic infection caused by the tapeworm *Echinococcus granulosus* [1, 2]. This parasitic disease is endemic in various parts of the world, including the Middle East, India, Africa, Australia, Turkey, Southern Europe, and other regions where sheep are raised. The disease is particularly prevalent in societies that have high contact with livestock. The life cycle of *E. granulosus* involves dogs as the definitive hosts and sheep or cattle as intermediate hosts [1, 2]. Humans become accidental hosts after they ingest viable eggs, either through direct contact with these animals or by consuming contaminated food or water. The tapeworm commonly affects the liver (60–70%) and lungs (20%) via the portal circulation, but it can invade almost every organ or tissue. In severe cases, this invasion can lead to serious complications such as anaphylaxis, infection, organ dysfunction, and potentially death [1].

Orbital hydatid cysts are rare, accounting for less than 1% of all hydatid cases [3]. These cysts can occur in any part of the orbit, with the intraconal space being the most frequently affected [4]. The most common clinical features of orbital hydatid cysts include slowly progressive unilateral proptosis, visual deterioration, periorbital pain, headache, and disturbances in ocular mobility.

Diagnosis of hydatid disease typically involves imaging techniques such as ultrasonography, computed tomography (CT) scan, and magnetic resonance imaging (MRI) [5]. In addition, serological tests like enzyme-linked immunosorbent assay (ELISA) or Western Blot may be utilized for confirmation in uncertain cases.

Treatment of hydatid disease can be complex and often involves substantial surgery and extended drug therapy. Complete surgical removal of the cyst is the mainstay of treatment, especially in cases with complications or when percutaneous drainage is impossible. Adjunctive medical treatment, including antiparasitic drugs such as benzimidazoles, is recommended in the postoperative period, particularly if the cyst ruptures intraoperatively or to prevent relapse [1].

Although hydatid disease is rare, it’s imperative for physicians, particularly those practicing in endemic regions, to understand its various presentations. This knowledge is essential due to the disease’s asymptomatic course and the potential for misdiagnosis, which could lead to unfavorable outcomes.

A mere handful of cases involving the extraocular muscles have been reported in the literature. In this report, we present an unusual case of an orbital hydatid cyst originating from the inferior rectus muscle, causing compressive optic neuropathy.

## Case presentation

### History and presentation

A 22-year-old male patient from Kabul, Afghanistan, presented himself to our oculoplastic clinic at Farabi Eye Hospital. He reported a five-month history of progressive proptosis in his left eye, which had been associated with a gradual decrease in vision over the past three weeks. The patient experienced very mild pain. He had a negative past medical history.

**Fig. 1:**
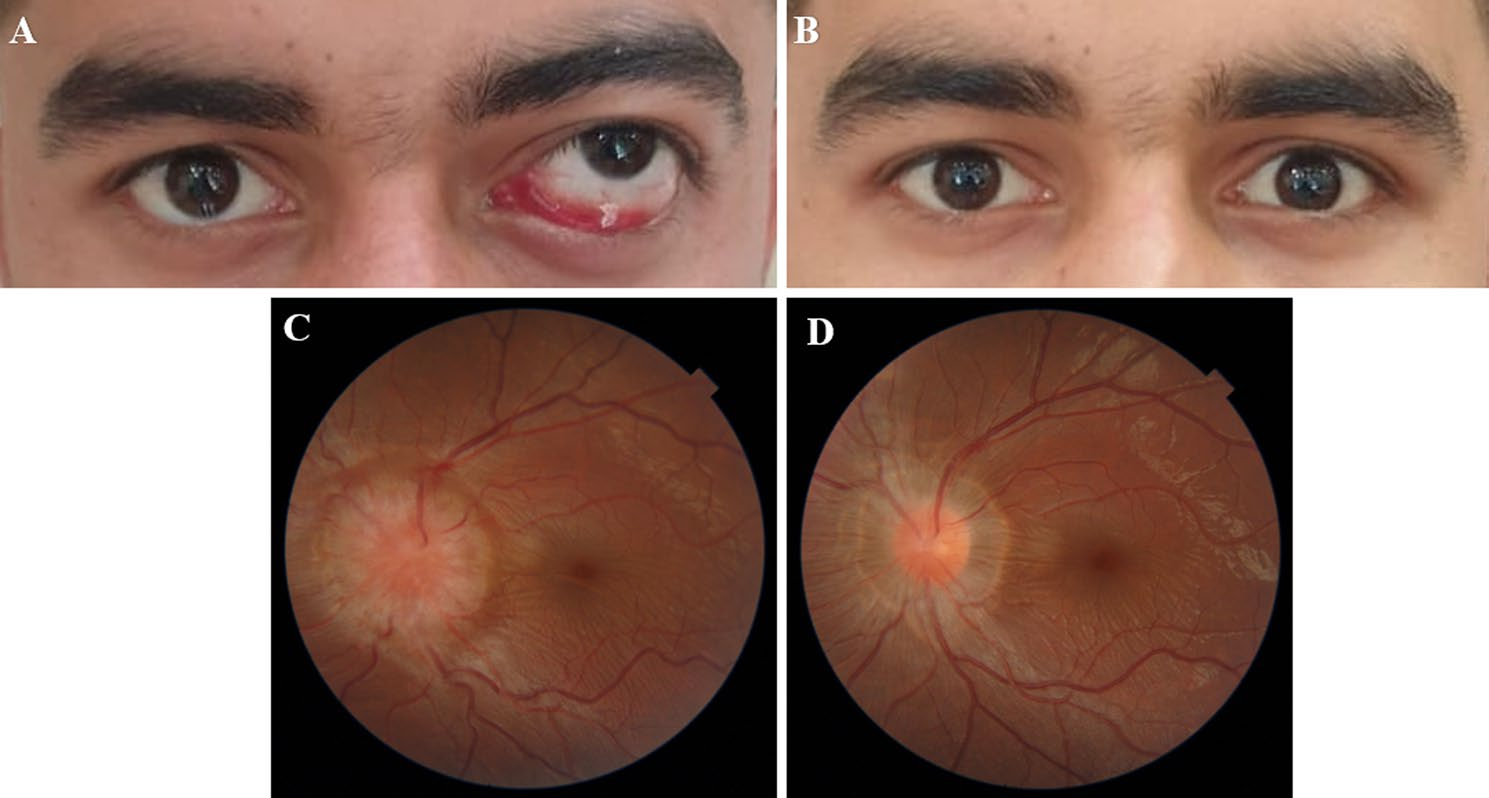
**A**) Depicts the preoperative image exhibiting the patient’s left globe in a state of superior dystopia, accompanied by chemosis and restricted movement. (**B**) Displays the postoperative image showing the fully resolved conditions. (**C**) Illustrates a grade 5 optic nerve head swelling. (**D**) Demonstrates a significant improvement of optic disc swelling observed following the cyst’s removal

Upon ophthalmic examination, the patient’s visual acuity was 20/20 in the right eye and 20/200 in the left eye. The left eye exhibited proptosis, upward globe dystopia, ocular motility limitation in all gazes, mild conjunctival injection, and mild chemosis (Fig. 1A). The fundus examination showed severe disc swelling (Frisen grade 5) (Fig. 1C). A relative afferent pupillary defect of 2 + was noted. Exophthalmos, measured using the Hertel exophthalmometer, was 17 mm in the right eye and 21 mm in the left eye. The intraocular pressure (IOP) was 17 mmHg in both eyes. The examination of the right eye yielded normal results. The patient’s medication history included amoxicillin/clavulanate, prednisolone, and metronidazole, which were previously prescribed by another physician.

**Fig. 2:**
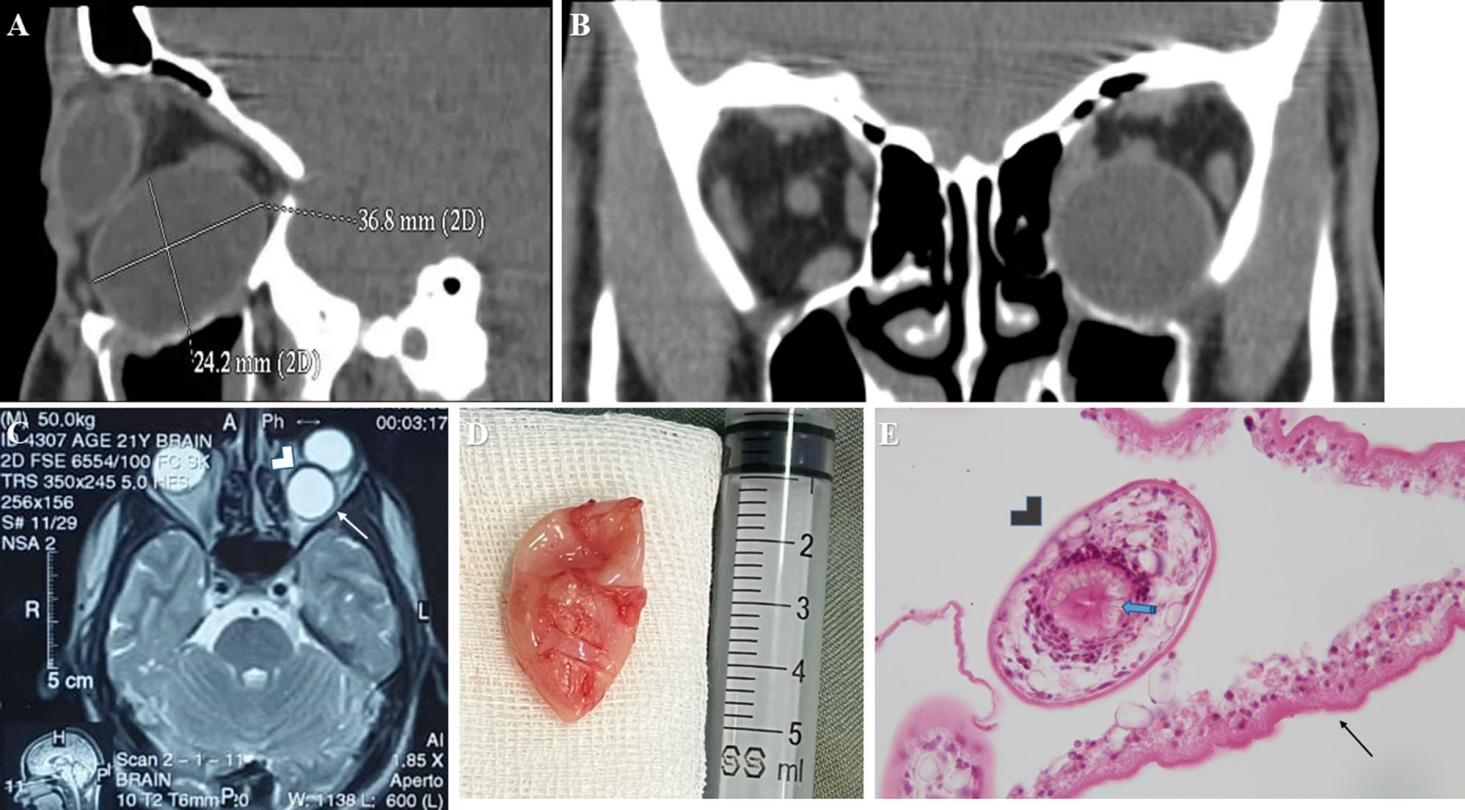
(**A**) This panel presents a sagittal plane computed tomographic (CT) scan of the left orbit. The scan reveals a cyst originating from the inferior rectus muscle, exerting pressure on the optic nerve, and displacing it upward. (**B**) A retrobulbar coronal plane CT scan illustrating the cyst in the inferior rectus muscle. (**C**) This panel features an axial plane MRI of the brain and orbit. The T2-weighted sequence reveals a hyperintense, well-defined, lesion (arrow) with a thick wall (arrowhead). (**D**) This panel illustrates the cyst after a three-step procedure: initial aspiration to decrease the cyst’s volume, followed by complete resection, and finally extensive irrigation to eliminate any remaining cystic components. (**E**) This pathology slide displays a germinal membrane (arrow) and protoscolices (arrowhead), which include a sucker and refractile hooklets (blue arrow), magnified 400 times

A paranasal-orbital CT scan revealed a large, oval, well-defined, thin-walled, homogenous cystic mass measuring approximately 36 × 25 mm (Fig. 2A). This mass was positioned inferior to the globe in the left inferior rectus muscle (Fig. 2B). It was observed to compress the optic nerve near the apex of the orbit, causing significant upward globe displacement. No bone erosion was detected. An orbital MRI showed that the cystic lesion was hypointense on T1-weighted images and hyperintense on T2-weighted images, with marginal ring enhancement following contrast injection (Fig. 2C). The patient’s laboratory tests, including a complete blood count and thyroid function tests, were within normal limits.

### Surgical technique

An inferior transconjunctival orbitotomy was initiated by creating an incision 4 mm below the inferior tarsal border and dissecting towards the inferior orbital rim. Further dissection was performed in a pre-periosteal plane to gain access to the cyst, which was embedded within the fibers of the inferior rectus muscle. Given the large size of the cyst, which prevented further exposure, needle aspiration of the cystic fluid was performed as an initial step, during which a total of 15 cc were aspirated. Then, the fibers of the inferior rectus muscle were then carefully displaced to facilitate direct access to the cyst. The cyst wall was delicately excised, ensuring the preservation of its integrity (Fig. 2D). Post-excision, the surgical field was irrigated with a hypertonic saline solution. Hemostasis was achieved, and the conjunctiva was sutured using 8 − 0 Vicryl. An antibiotic ointment was applied to the eye, and the excised specimen was sent for histopathological evaluation. There were no post-op complications.

### Postoperative course and follow-up

The pathology slides and cytology of the hydatid cyst biopsy revealed a cystic structure, evident in the hematoxylin and eosin stain slides (Fig. 2E). This structure comprised an outer acellular eosinophilic laminated membrane connected to a transparent germinal membrane, which was nucleated. The germinal membrane exhibited budding protoscolices. Additionally, a daughter cyst with protoscolices, identifiable by its sucker and refractile hooklets, was observed. The cytology smear from the cyst aspiration further confirmed the presence of these protoscolices.

The patient was discharged one day after the surgery with a prescription for systemic albendazole (15 mg/kg/day, divided into two doses) to be taken over 28 days. Subsequently, a chest CT scan and abdominal ultrasonography showed no suspected lesions in either the chest or abdomen.

One-week post-operation, the patient exhibited complete resolution of the proptosis, and his vision improved to 20/30 (Fig. 1B). Ocular motility showed significant improvement, with a residual diplopia only in the downgaze. After one month, the patient’s vision further improved to 20/20, there was a -2 limitation supraduction, and the optic disc swelling had significantly resolved to a Frisen grade 1 (Fig. 1D). By 3 months post-operation, there was no limitation in ocular motility. During a telephonic follow-up conducted 10 months post-surgery, the patient reported experiencing no abnormalities. The patient will continue to be monitored on a yearly basis to inspect for any signs of recurrence.

## Discussion and conclusions

Hydatid disease, a zoonotic infection caused by the larval stage of the tapeworm Echinococcus granulosus, is a significant global health issue, especially in livestock-rich regions like the Middle East, including Iran. The disease, primarily affecting the liver and lungs, can infest almost any organ or tissue in the human body, including the head and neck, orbit, and central nervous system, through the ingestion of ova from definitive hosts like canines. A notable yet rare manifestation of this disease is orbital hydatid disease, often seen in children and young adults. These cysts, typically located in the retrobulbar region, either extraconally or intraconally, have a slight predilection for the left orbit, possibly due to the path of the left carotid artery [6].

Table 1 provides a summary of the unique cases of orbital hydatid cysts, specifically those involving the extraocular muscles, as reported in the literature. A total of five such cases have been documented between the years 2003 and 2023 ^4, 7–10^. The first of these cases originated from Turkey, while the subsequent four were all reported from Iran. When considering our case as well, the average age of the patients is 25 years, ranging from 12 to 39 years. The occurrence is evenly distributed between males and females. Four of these cases involved the inferior rectus muscle, all on the left orbital side [7–9]. Additionally, two cases involved the medial rectus muscle, both on the right side [4, 10]. The surgical interventions in all these cases were successful, with no recurrence reported. No instances of spontaneous cyst rupture were observed in any of the cases. Our case is notable as it is the fourth reported instance involving the inferior rectus muscle. Moreover, it is the first case that presented with compressive optic neuropathy. Fortunately, this condition was resolved, and the patient regained full visual function postoperatively.

**Table 1:** Case reports of orbital intramuscular hydatid cysts

Author	Country	Age (year)	Sex	Laterality	Location	VA	Clinical feature	Surgical Management	Medication	Outcome
Kirati et al. [7]	Turkey	20	F	R	MR	20/20	Painful eye movements, blurred optic disc	Medial transconjunctival, cyst removed completely. Irrigation with hypertonic saline for 15 min	Albendazole 400 mg twice daily, 2 cycles	No recurrence
Rajabi et al. [4]	Iran	24	M	R	MR	20/20	Proptosis	Medial orbitotomy, trans caruncle incision	Albendazole 10 mg/kg daily for 12 weeks	No recurrence
Saravi et al. 2016	Iran	12	F	L	IR	20/100	Painless proptosis, nausea, vomiting, productive cough	Inferior orbitotomy, cyst removed completely	Albendazole (10 mg/kg/d) for 12 weeks	No recurrence
Rajabi et al. [8]	Iran	34	M	L	IR	-	Limitation in ocular motility, proptosis, and chemosis	Inferior trans-conjunctival incision, content aspirated, irrigated by a 15% hypertonic saline, and re-aspirated; cyst was resected completely	Albendazole (400 mg twice daily) and Praziquantel (40 mg/kg per day) 2 weeks before operation and continued 3 months thereafter	No recurrence
Ghaedamini et al. [9]	Iran	39	F	L	IR	20/20	Hyperglobus, limitation of infraduction and conjunctival injection	Inferior transconjunctival orbitotomy	Albendazole and praziquantel for 3 months	No recurrence
Current case 2024	Iran	22	M	L	IR	20/200	Progressive proptosis, decreased vision, dystopia, motility limitation, mild injection and chemosis	Inferior trans-conjunctival orbitotomy, needle aspiration then complete excision, saline irrigation	Albendazole (15 mg/kg/day, divided into two doses) for 28 days	VA: 20/20 No recurrence

VA: visual acuity; F: female; M: male; R: right; L: left; MR: medial rectus muscle; IR: inferior rectus muscle

The clinical manifestations of hydatid cysts primarily result from their mass effect on adjacent structures, especially in confined spaces like the orbit. The most common presentation of intraorbital hydatid cyst is a slow-growing, unilateral proptosis, which can be axial or non-axial, painless, irreducible, non-pulsatile, and non-blowing [11]. If the cyst cracks, it can become inflamed. Other symptoms include ocular pain, diplopia, headache, blurred vision, visual loss, chemosis, palpebral edema, restriction of extraocular movements, and orbital cellulitis. Later manifestations might include optic disc swelling, and optic atrophy with consequent abnormal papillary defect, retinal vein engorgement, erosion of orbital bone, hypopyon, and eyelid edema [4, 10, 12]. The severity of symptoms depends on the parasite load as well as the site and size of the cyst. The cysts grow at an average rate of about 1–1.5 cm per year, and due to the limited space in the orbital cavity, patients usually become symptomatic within two years [4, 13]. In the literature, while a definitive criterion for a ‘giant’ orbital hydatid cyst is yet to be established, cysts larger than 5 cm have been referred to as such. For instance, Kumar et al. reported a ‘giant’ orbital hydatid cyst measuring 6.8 cm [14]. In contrast, the cyst in our case study measures 3.6 cm.

Diagnosis of orbital intramuscular hydatid cysts combines clinical findings, lab data, and imaging studies. Histopathological examination typically confirms the diagnosis [1, 4]. Differential diagnoses include dermoid and epidermoid cysts, teratoma, inflammatory pseudotumor, abscesses, hemangiomas, post-traumatic hematomas, mucocele, and schwannoma [15]. For instance, abscesses often show orbital fat stranding and central diffusion restriction, frequently associated with sinusitis. Dermoid cysts typically present as a slow-growing, non-tender mass near the lateral eyebrow. Inflammatory pseudotumors are characterized by sudden onset of pain, proptosis, and erythema, with pain intensifying during extraocular muscle movement. On CT scan, hydatid cysts appear uniloculated, homogeneous, and can be hypo or hyperdense to vitreous, potentially causing orbital bone thinning. They are often located in the superolateral and superomedial angles of the orbit. MRI is useful for evaluating the cyst’s inner structure and orbital soft tissue involvement. Following the administration of contrast, both CT scan and MRI show peripheral rim enhancement [5]. On MRI, the cysts reveal low signal intensity on T1-weighted images and high signal intensity on T2-weighted images, which may vary with the hydatid cyst’s development phase (viable/infected/dead). Notably, hydatid cysts exhibit a thicker pseudocapsule around the cyst than other orbital cysts. Ultrasound typically shows a “double layer sign”. Histopathological examination often provides the definitive diagnosis, demonstrating an eosinophilic acellular laminated structure with an inner germinal layer and multiple protoscoleces infiltrating between the bony trabeculae [1].

The management of orbital hydatid disease is primarily surgical, with the goal being the complete excision of the cyst without rupture. This is often challenging due to the complex anatomy of the orbit and the fragility of the cyst’s wall. If a hydatid cyst is suspected, preoperative treatment with anthelmintic drugs, particularly albendazole, is crucial to prevent parasite seeding and anaphylactic reactions in case the cyst ruptures during surgery. This treatment typically lasts for 2 to 4 weeks.

The surgical approach depends on the size and location of the cyst, its extension into the cranial cavity, and the surgeon’s expertise and preference. Extra-cranial approaches, such as lateral orbitotomy, trans-maxillary inferior orbitotomy, and supraorbital eyebrow incision orbitotomy, are commonly used for cysts confined within the orbit. Trans-cranial approaches, such as subfrontal, pterional, and fronto-orbito-zygomatic, are suitable for lesions extending into the cranium. In cases where complete resection is impossible due to adhesion, the approach may be changed to cyst disruption, membrane removal, and infusion of scolicidal hypertonic saline. This is done to prevent the spread of parasitic disease. In the event of a cyst rupture, copious irrigation with hypertonic saline is performed. This destroys the scolices in the operative field by osmotic desiccation. The anesthesiologist should be promptly informed to take measures like administering antihistaminics and dexamethasone to prevent anaphylaxis. While surgery remains the traditional treatment of choice, the PAIR method (Puncture, Aspiration, Injection, Re-aspiration) has emerged as a minimally invasive therapeutic option for intra-abdominal localizations of hydatid cysts. However, for orbital hydatid cysts, surgical removal is still the best option. Bagheri et al. have reported some success with the PAIR method in orbital hydatid cysts, reinforcing that surgical removal is the superior option [16]. Sendul et al. utilized a medial transconjunctival orbitotomy approach for a hydatid cyst, necessitating the intentional rupture of the cyst due to the anatomical constraints of the surgery area [17]. This highlights the inherent challenges when dealing with medially located cysts. Postoperative treatment with albendazole or mebendazole is recommended to reduce the risk of relapse. Albendazole is preferred due to its better systemic absorption and penetration inside the cyst. This treatment also decreases the risk of relapse.

Preventing hydatid disease requires a comprehensive approach, including basic hygiene practices like handwashing after dog contact and vegetable cleanliness. Regular dog deworming with praziquantel, improved livestock slaughter hygiene, and public education can reduce transmission significantly [18]. The E. granulosus recombinant antigen (EG95) vaccine in sheep has shown promise [1]. A program combining lamb vaccination, dog deworming, and older sheep culling could potentially eliminate human cystic echinococcosis within a decade. Additional measures include stray dog control, home livestock slaughter restrictions, preventing dogs from feeding on infected sheep carcasses, and teaching children hand hygiene. Implementing these prevention strategies faces significant challenges. Dog treatment campaigns often struggle with coverage, sensitivity, and high costs. The ELISA test’s high costs and need for specialized equipment limit its use, especially in endemic areas with limited resources [18]. While sheep vaccines show promise, they require enhancements for broader protection and data on longevity. The feasibility of large-scale production and the effectiveness of E. granulosus dog vaccine candidates when administered orally have not been determined. The potential of a combined rabies/echinococcal oral vaccine is significant, but its effectiveness remains unproven [18].

This case report details an unusual instance of an orbital intramuscular hydatid cyst causing compressive optic neuropathy. Despite its rarity, the case emphasizes the importance of including hydatid disease in the differential diagnosis of orbital masses, particularly in endemic regions. It underscores the need for a comprehensive management approach, combining surgical intervention and adjunctive medical treatment. The report also highlights the role of preventive measures in curbing the transmission of this zoonotic infection. This article contributes to the limited literature on orbital hydatid cysts involving extraocular muscles, and provides valuable insights into the clinical presentation, diagnosis, and management of this condition.

## Data Availability

The datasets used and/or analyzed during the current study are available from the corresponding author on reasonable request.

## Abbreviations

CT: Computed tomography
MRI: Magnetic resonance imaging
ELISA: Enzyme-linked immunosorbent assay
IOP: Intraocular pressure
